# Infantile Spasms: An Update on Pre-Clinical Models and EEG Mechanisms

**DOI:** 10.3390/children7010005

**Published:** 2020-01-06

**Authors:** Remi Janicot, Li-Rong Shao, Carl E. Stafstrom

**Affiliations:** Division of Pediatric Neurology, The Johns Hopkins University School of Medicine, Baltimore, MD 21287, USA; rjanico1@jhmi.edu (R.J.); lshao5@jhmi.edu (L.-R.S.)

**Keywords:** infantile spasms, West syndrome, epilepsy, childhood, epileptic encephalopathy, electroencephalogram (EEG), hypsarrhythmia, electrodecrement, animal model

## Abstract

Infantile spasms (IS) is an epileptic encephalopathy with unique clinical and electrographic features, which affects children in the middle of the first year of life. The pathophysiology of IS remains incompletely understood, despite the heterogeneity of IS etiologies, more than 200 of which are known. In particular, the neurobiological basis of why multiple etiologies converge to a relatively similar clinical presentation has defied explanation. Treatment options for this form of epilepsy, which has been described as “catastrophic” because of the poor cognitive, developmental, and epileptic prognosis, are limited and not fully effective. Until the pathophysiology of IS is better clarified, novel treatments will not be forthcoming, and preclinical (animal) models are essential for advancing this knowledge. Here, we review preclinical IS models, update information regarding already existing models, describe some novel models, and discuss exciting new data that promises to advance understanding of the cellular mechanisms underlying the specific EEG changes seen in IS—interictal hypsarrhythmia and ictal electrodecrement.

## 1. Introduction

Epileptic encephalopathies (EEs) are a spectrum of disorders that mostly begin during infancy and have poor neurological and behavioral outcomes. According to the International League Against Epilepsy definition, an EE is defined as a syndrome in which seizures or interictal epileptiform activity contribute to or worsen brain dysfunction, above and beyond what might be expected from the underlying pathology alone [1]. EEs exact an immense toll on children, their families, the health care system, and society. Unfortunately, affected patients suffer from ongoing seizures and cognitive dysfunction, despite extensive pharmacological treatment. These observations emphasize the need to develop novel treatments, a circumstance that is dependent on clarifying the pathophysiology through pre-clinical (usually animal) models [2].

West syndrome is an EE with a number of features distinct from other epilepsies, including a characteristic seizure semiology (flexion or extension spasms—infantile spasms (IS)), age-specific onset in the middle of the first year of life (peak~six months of age), unique interictal and ictal electroencephalogram (EEG) findings, response only to certain treatments, and cognitive or behavioral stagnation or decline. For simplicity here, and in keeping with the recent literature, we refer to West syndrome as IS, acknowledging that the broader term “epileptic spasms” can be applied to the spasm seizure semiology at any age. This concept is relevant here, because, in some animal models discussed below, spasms occur outside the equivalent age range during which IS occur in humans.

The interictal EEG of IS is called hypsarrhythmia and it consists of chaotic, high amplitude, mixed slow, and sharp wave activity, while the ictal EEG signature during an actual spasm is a generalized voltage attenuation (electrodecrement), sometimes combined with very fast oscillations (VFOs; typically >70 Hz) [3,4]. The spasms in IS often occur in clusters during sleep state transitions. IS and the underlying interictal EEG pattern (hypsarrhythmia) often respond to the stress hormone adrenocorticotrophic hormone (ACTH) [5] or corticosteroids, but not to most conventional antiseizure drugs (ASDs), which further underscores the uniqueness of this disorder. The fact that the initial documentation of ACTH responsiveness in IS was serendipitous [5] is relevant to the ongoing search for mechanisms and therapies.

IS affect 2–5/10,000 infants, which makes it the most frequent epilepsy beginning during the first year of life [6,7]. IS occurrence within this specific age window has definite mechanistic implications. The etiology of IS can be symptomatic (known cause) or cryptogenic (no known cause), though with advances in genetics, imaging, and other diagnostic tools, the proportion of cryptogenic cases is decreasing over time. IS can occur in full term infants, as well as those born prematurely (in which case the most common associated causes are periventricular leukomalacia and intraventricular hemorrhage) [8,9]. The outcome of IS is usually poorer in symptomatic cases. The causes of IS are heterogeneous—over 200 etiologies have been linked to IS, spanning the spectrum from acquired brain injury (e.g., perinatal hypoxia ischemia, intracranial hemorrhage) to mutations in genes regulating the development of ion channels, synaptogenesis, neuronal migration, and circuit formation [10,11,12]. The current first-line treatments include ACTH, corticosteroids, and vigabatrin; the ketogenic diet has also shown some efficacy [13,14,15]. The usual response to treatment with ACTH or corticosteroids is “all-or-none”—the spasms and hypsarrhythmia subside, or no improvement is seen at all. This observation needs to be considered when explaining the mechanism of action of these agents in IS. However, these drugs are ineffective in at least 30% of patients and they are associated with potentially severe side effects [16,17,18]. It is possible that trials combining multiple mechanisms of action (e.g., ACTH plus vigabatrin) might be beneficial in IS, and animal studies could be used to screen such potential therapies. However, to date, such combination therapy has not been shown to significantly improve developmental outcome [19]. The poor prognosis of patients with IS and the inadequacy of current therapies pose great challenges to clinicians and highlight the dire need for a better understanding of the syndrome to create more potent treatments. At present, the pathophysiology of IS remains elusive.

## 2. Preclinical Studies of Infantile Spasms—Animal Models

The rarity of IS and the complexity of human brain development make it challenging to undertake large scale clinical studies. Numerous genes have been linked to IS, spanning an incredibly diverse spectrum of molecular and cellular mechanisms, which somehow converge onto a relatively similar clinical presentation. Over the past 15 years, several animal models have been developed to gain better understanding of the pathogenesis of IS with the goals of developing novel, mechanism-based treatments, and improving neurological outcome.

**Figure 1:**
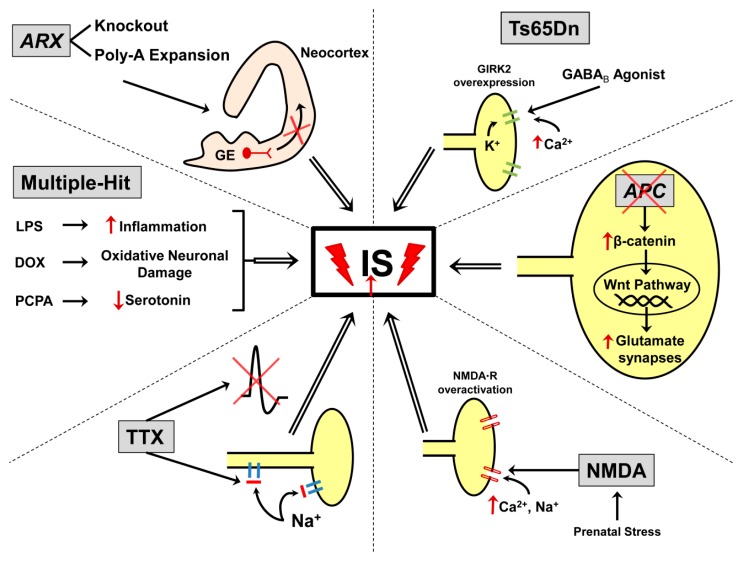
Sites of potential pathophysiology of IS in selected animal models. Neuronal somata and axons are shown in yellow. Top left, Selective mutation of ARX (knockout or knock-in (poly-alanine expansion)) from cortical interneurons leads to abnormalities of GABAergic interneuron migration and function. Top right, In the Ts65Dn Down syndrome model, there is dysfunction of the inward rectifying potassium channel, GIRK2, which might allow excessive Ca^2+^ influx and hyperexcitability; GABA_B_ receptor agonists induce spasms in this model. Middle right, Conditional deletion of APC leads to increased β-catenin levels, increased number of glutamatergic synapses, and development of IS. Bottom right, Prenatal stress (such as immobilization stress or betamethasone exposure) alters expression of genes involved in excitatory and inhibitory synaptic function; postnatal injection of NMDA causes hyperactivation of glutamate receptors and increased Ca^2+^ influx. Bottom left, TTX infusion blocks Na^+^ channels of both axons and somata, inhibiting neuronal firing in neocortex, which becomes essentially deafferented by this drug; spasms then begin in the hemisphere contralateral to the TTX injection. Middle left, Multiple-hit model uses the combination of the antineoplastic drug DOX, the pro-inflammatory agent LPS, and the serotonin-depleting compound PCPA to induce large cortical structural lesions, replicating some features of severe symptomatic IS. See text for details. Abbreviations: IS, infantile spasms; GE, ganglionic eminence; ARX, Aristaless-related homeobox gene; poly-A, poly-alanine; GIRK2, G-protein coupled rectifying potassium channel type 2; GABA_B_-R, γ-aminobutyric acid receptor type B; APC, adenomatous polyposis coli; NMDA-R, N-methyl-D-aspartate receptor; Ca^2+^, calcium; Na^+^, sodium; K^+^, potassium; TTX, tetrodotoxin; DOX, doxorubicin; LPS, lipopolysaccharide; PCPA, p-chlorophenylalanine.

In this review, we focus on the pathogenic mechanisms and therapeutic responses of IS animal models (Figure 1), each of which meets some of the criteria set forth initially by Stafstrom & Holmes in 2002 (Table 1) [20]. It is important to keep in mind species-specific differences in brain development when evaluating the various models (and hence in seizure susceptibility) [21]. As a broad generalization, rodent postnatal day (P) 7 approximates human brain development in the late preterm phase, P10 reflects term human brain status, and ~P14-21 might best approximate human ages at which IS begin [22,23]. Each model is valuable for investigating particular pathways or cellular events involved in IS, while no model should be expected to replicate all of the phenotypic features of human IS (Table 2). This article provides a summary updating pre-clinical models of IS; the reader is referred to prior reviews of this topic for additional details [24,25,26,27]. We also describe recent progress in dissecting the pathophysiology of the characteristic EEG findings in IS, which has been a neglected area of research until recently [4].

### 2.1. Genetic Models

#### 2.1.1. Decreased GABAergic Inhibition: *ARX* Mutations

##### *Arx* Knockout Model

The Aristaless-related homeobox gene (*ARX*) is a transcription factor that primarily acts as a transcriptional repressor in regulating the specification and migration of interneurons from the forebrain ganglionic eminences to the neocortex [28]. *ARX* affects the transcription of more than 80 downstream genes [29]. The mutations in *ARX* have a well-established correlation with multiple types of neurodevelopmental and epileptic disorders, including IS [30]. The disruption of inhibitory GABAergic systems (termed “interneuronopathy”) has been linked to several epilepsies [31].

Complete knockout of *Arx* from cortical interneurons of mice engenders severe interneuron migration irregularities, often leading to perinatal death [32]. However, the conditional deletion of *Arx* from cortical interneurons of either male or female mice leads to IS-like spasms with EEG electrodecrements, followed by various seizure phenotypes in adulthood [24]. More recently, the targeted knockout of *Arx* from interneurons prior to their migration from the ventral forebrain to dorsal neocortex resulted in a decrease of all interneuron subtypes, which supports the role of *Arx* in interneuron migration and the idea that IS in such mice (and humans) could be a result of developmental disinhibition [33].

While no treatments have been reported using this model, it does provide information regarding the pathogenesis of IS and strengthens the link between interneurons and different types of epilepsy, including IS.

##### *Arx* Expansion Model

While the mouse *Arx* knockout model is useful for studying the development of IS, most *ARX* mutations in humans are expansions, not deletions. Such expansions involve the first polyalanine tract of the protein [34]. The mice with this *Arx* expansion (*Arx*^(GCG)10+7^) develop myoclonic seizures early in life and other spontaneous seizures in adulthood. EEGs on pups show multifocal spikes as well as electrodecrements during spasms, like humans. Histological studies of these mutant mice show a reduction of *Arx* in calbindin, neuropeptide Y, and cholinergic interneurons, while having no effect on parvalbumin- or calretinin-expressing interneurons [35], suggesting that specific inhibitory pathways may have key functions in the development of IS (contrasted with the knockout model).

17β-estradiol was administered to neonatal (P3–10) animals, which led to transcriptional changes and re-established functional inhibitory pathways, in an attempt to restore GABAergic function in this model [36]. Phenotypically, neonatal spasms as well as adulthood seizures were suppressed by 17β-estradiol treatment, and there was restoration of depleted interneuron populations. Endogenous estrogen levels in mice surge between embryonic day 9 and postnatal day 10 (equivalent to full term human), which correlates with a critical period for interneuron migration and partly explains the efficacy of this treatment in treating spasms in this model. It must be noted that estradiol does not decrease spasms in other IS models (see Section 2.2.3 and Section 2.2.4).

Although *ARX* mutations are a rare cause of IS in humans, *Arx* mouse models are important, because they allow for a genotype-phenotype correlation with specific and relevant pathophysiology and they are amenable to the testing of existing and novel therapies [30].

#### 2.1.2. Excessive GABA_B_ Receptor-Mediated Potassium Currents: Ts65Dn Down Syndrome Model

Children with Down syndrome are at high risk for developing IS [37]. A mouse model of Down syndrome, called Ts65Dn, has been studied to provide insights into the pathogenesis of IS in Down syndrome. GABA_B_ receptor agonists elicit seizures in several animal models. In Ts65Dn mice, the injection of GABA_B_ receptor agonists, such as baclofen or gamma-butyrolactone, leads to extensor spasms associated with ictal spikes and electrodecrements that were abolished by vigabatrin or ACTH_1–24_ administration [38]. Ts65Dn mice overexpress the G-protein-coupled inward rectifying potassium channel subunit 2 (GIRK2) [39], which increases postsynaptic GABA_B_ currents in brain slices that were prepared from Ts65Dn mice [40]. It is unknown how such increased GABA_B_ activity leads to hyperexcitability, but the data indicate that the mutated GIRK2 causes the channel to lose its ion selectivity—in addition to altering K^+^ efflux, mutations in GIRK2 allow for excessive Ca^2+^ influx, providing a plausible basis for increased excitability [41].

The overexpression of GIRK2 is necessary for the production of the IS-like phenotype, as the knockdown of the *Kcnj6* gene (which codes for GIRK2) made the mice resistant to GABA_B_ agonist-induced spasms [42,43]. However, trisomy of Kcnj6 is not sufficient for replicating the desired phenotype. Instead, the overexpression of this G-protein subunit is likely one of several brain alterations in Ts65Dn mice that somehow precipitates IS [44]. The mechanisms that alter excitation/inhibition balance and lead to heightened susceptibility to spasm-like seizures in the Ts65Dn mouse are unknown, but a postsynaptic GABA_B_ receptor localization is hypothesized [40]. It is possible that other ion currents are altered in this model, as cultured neurons from Ts65Dn mice display abnormalities in a variety of potassium channels and hyperpolarization-activated cation (HCN) channels [45]; typically, a decrease in HCN current increases the cellular excitability. Importantly, those experiments were not performed on the IS model (no GABA_B_ receptor agonist stimulation) [45]. The full pathophysiology that underlies spasm-like seizures in this model remains to be determined.

While the Ts65Dn model is only applicable to IS in patients with Down syndrome, it does illustrate the importance of GABA_B_ receptor and potassium channel regulation in the pathogenesis of IS. The circuitry mediating IS in Ts65Dn mice is unknown, but the involvement of brainstem networks is hypothesized [38,39].

#### 2.1.3. Increased Synaptic Excitation: APC Conditional Knock-out

β-catenin is a cadherin adhesion complex that is critical for many aspects of normal brain development, including the Wnt signaling pathway [46]. The levels of β-catenin are tightly regulated by several genes, including *APC* (adenomatous polyposis coli). The mutation of *APC* leads to excessive β-catenin, causing abnormal dendritic branching and an increase in the number of excitatory synapses [47]. Interestingly, APC is an mRNA-binding protein that negatively regulates β-catenin and it interacts with at least five different gene products (*Foxg1*, *LIS1*, *STXBP1*, *DCX*, and *NR2F1*) that are involved in IS [11].

The conditional deletion of *APC* from CamKIIα positive neurons, which play a significant role in glutamatergic neuron development, leads to IS-like features in neonatal mice [48]. The mice exhibit flexion-extension spasms with abnormal EEGs from P5–P14, which develop into spontaneous seizures in adulthood. In *APC* knockout mice, there is an increase in the number of glutamatergic layer 5 pyramidal neurons, and these neurons have enhanced excitatory postsynaptic currents, affording a rational explanation for the increased seizure susceptibility. These pathophysiological observations are relevant in terms of the emerging understanding of the cellular changes underlying EEG changes in IS (see Section 3). Moreover, *APC* knockout mice display autistic-like features, including repetitive behaviors and reduced social interest [49]. Pharmacological interventions to alter the β-catenin/canonical Wnt signaling pathway in this model are not yet reported.

### 2.2. Acquired/Provoked Models

#### 2.2.1. Stress in the Developing Brain: Corticotropin-Releasing Hormone Model

In the developing brain, stress increases neuronal excitability and predisposes to seizures [50]. Stress hormones, ACTH and glucocorticoids, ameliorate IS [51]. The appreciation of the diverse and multiple etiologies of IS led to the hypothesis that stress in the developing brain is a common factor in the development of this syndrome [52]. Stress increases the release of an endogenous proconvulsant hormone, corticotropin-releasing hormone (CRH). Intraperitoneal or intracerebroventricular administration of CRH during the second week of life in rats causes severe seizures [53], whereas much higher doses of exogenous CRH are required for producing seizures at older ages. Therefore, excessive release of synaptic CRH acts as an endogenous convulsant in the developing brain. However, CRH-induced seizures are not spasms, but rather have a limbic semiology, possibly related to the abundant presence of CRH receptors in the amygdala and hippocampus. Acute ACTH treatment does not ameliorate seizures that are induced by exogenous CRH, but may suppress the level of endogenous CRH [54].

While CRH is necessary for the normal developing brain, excess CRH, as initiated by multiple stressors, results in seizures, dendritic, and neuronal structural abnormalities, and long-term cognitive, learning, and memory deficits [55]. These observations are relevant with respect to the cognitive deficits that are seen in children with IS. The CRH model does not entail structural brain damage, so any cognitive deficit will likely be due to the seizures, as consistent with the epileptic encephalopathy concept.

The limitations of the CRH model include the EEG findings (focal sharp waves rather than hypsarrhythmia and electrodecrement) and the lack of spontaneous seizures. Nonetheless, the CRH model provides insight into the age-specific effects of stress on seizure susceptibility and increases the understanding of ACTH and corticosteroid actions on brain development [56]. The hypothesis that stress heightens neuronal excitability is supported by recent evidence that, via an increase in CRH, chronic naturalistic stress (depriving the dam and pup of adequate bedding material) increases seizure susceptibility and, in some cases, promotes spasm-like clinical behavior and EEG changes [57]. The effects of CRH on neuronal excitability could provide a window into the basic mechanisms of hypsarrhythmia (see Section 3).

#### 2.2.2. Sodium Channel Blockade: TTX Model

Tetrodotoxin (TTX) is a sodium channel blocker that eliminates all neural activity. It was hypothesized that the chronic suppression of neural activity during specific time windows of brain development could lead to hyperexcitability or seizures (neuronal desynchronization hypothesis) [58]. IS-like flexion seizures were observed when TTX was infused into the neocortex or hippocampus via an implanted osmotic minipump in P10-12 rats. IS-like spasms developed about 10 days after TTX infusion and continued for days to weeks after the TTX pump was removed [59]. The existence of a latent period from TTX infusion until spasms occurrence mimics the human condition. On EEG, the chronic infusion of TTX was associated with high amplitude, low frequency waves followed by electrodecrement, patterns that are quite reminiscent of hypsarrhythmia, as seen in humans. It is unknown whether TTX injections into other brain sites or at other ages would produce similar changes.

The presence of high-frequency oscillations (HFOs), occurring during ictal events on the side contralateral to the TTX infusion, is a particularly interesting feature of this model [60,61]. Oscillations with frequencies of 250–600 Hz represent the synchronous firing of neuron populations and correlate with the sites of ictogenesis [62].

Not only do the EEG features in this model resemble those seen in IS patients, the response to ASDs is also similar. In a dose-dependent fashion, ACTH eliminated spasms in 66% of animals in this model only at the highest dose tried (32 IU/kg/day), and also attenuated the abnormal interictal EEG pattern [63]. Vigabatrin, which is used for IS in children with tuberous sclerosis and other etiologies, suppressed or delayed the onset of spasms and significantly reduced HFOs [64].

The electrographic similarities between this model and patients with IS are striking, providing a valuable tool for studying the fundamental neurophysiological basis of IS. Somatosensory cortex slices from TTX-treated rats with IS demonstrated network hyperexcitability with longer and more frequent HFOs [65]. This model is amenable to an investigation of mechanisms of hypsarrhythmia and electrodecrement (see Section 3). One limitation of this model is the age at which spasms occur and drugs work—rats start to have spasms at P35–40 (i.e., juvenile age), which is older than humans with IS.

#### 2.2.3. Increased Glutamate Receptor Activation: Prenatal Stress/NMDA Model

Intraperitoneal injection of the glutamate receptor agonist N-methyl-D-aspartate (NMDA) to rats induce flexion spasms that are associated with electrodecrement and chaotic interictal waves, leading to this IS model. The spasms and characteristic EEG behavior only occurred in young animals (P10–P15) and they were not stopped by pretreatment with conventional agents used to treat IS [66]. The lack of effect of treatment with corticosteroids was a major limitation of this model. However, by exposing rats to prenatal stress (betamethasone injection into the dams), NMDA-induced spasms in the postnatal animals became responsive to ACTH [67]. Prenatal acute immobilization stress or forced cold water swim stress also sensitized rat pups to NMDA-induced spasms, possibly by downregulating GABAergic systems and decreasing the expression of the potassium-chloride co-transporter 2 (KCC2) [56,68]. KCC2 is mainly responsible for establishing the chloride gradient by keeping intracellular chloride concentration low, so KCC2 reduction favors less hyperpolarization during GABAergic neurotransmission (and, hence, increased seizure propensity) [69]. It has been shown recently that calpain, a calcium-activated protease that is involved in excitotoxicity, can render GABAergic neurons excitatory by increasing the dephosphorylation and cleavage of KCC2 [70]. Moreover, the administration of a calpain inhibitor resulted in fewer NMDA-induced spasms in prenatally stressed rats, suggesting a potential novel treatment approach.

Perinatal stress, such as corticosteroid exposure, is thought to alter hypothalamus-pituitary-adrenal (HPA) axis responses, partially explaining the efficacy of hormonal treatment. For example, *in utero* betamethasone exposure significantly downregulates the transcription of genes critical for GABAergic and glutamatergic synaptic transmission in the hypothalamus. Sex-specific transcriptional patterns were identified, similar to the clinical situation [71].

Using this model, the efficacy of several compounds has been tested. In contrast to the *Arx* expansion model, neonatal 17β-estradiol failed to provide protection from NMDA-induced spasms [72]. The successful treatment of NMDA-induced spasms with ACTH and vigabatrin [73] suggests that both steroid hormones and GABAergic inhibition play a critical role in IS generation. Pretreatment with ganaxolone, which is a synthetic neurosteroid hormone and GABA_A_ enhancer, delayed the onset and reduced the number of spasms [74]. The ketogenic diet might also be efficacious in treating IS—in the NMDA model, prolonged pretreatment with the ketone body beta-hydroxybutyrate reduced the frequency of spasms and increased the latency period in addition to improving memory function in rats [75].

Evidence linking epilepsy and neuroinflammation has grown over the past decade, providing another potential therapeutic target in IS [76]. PMX53 is a potent inhibitor of the complement factor 5a receptor (C5ar1) and it has shown promise in epilepsy models [77]. Inhibiting this receptor during status epilepticus reduced tumor necrosis factor alpha (TNF-α) [78], a major inflammatory cytokine, which, along with interleukin 1 beta (IL-1β), initiates the immune response in the CNS, leading to neuronal damage and hyperexcitability [79]. In the NMDA IS model, the transcription of approximately 30% of hypothalamic genes was altered, with significantly greater changes in males. ACTH and PMX53 both restored these transcriptional changes back to control levels [77].

Hormonal treatment prevails as the leading therapy, despite all new therapeutic targets currently being investigated. However, ACTH is only partially effective and it carries potentially severe side effects. AQB-565 is pharmaceutically engineered fusion peptide containing the first 24 amino acids of ACTH and is a melanocyte-stimulating hormone analog. AQB-565 selectively interacts with CNS melanocortin receptors MC3 and MC4, in addition to suppressing spasms to the same extent as ACTH in the NMDA model [80]. The specificity of this treatment might offer the same benefits as ACTH, but with fewer side effects.

The NMDA model recapitulates some major clinical features of IS (i.e., EEG correlates and responsiveness to ACTH). A prior limitation was that in most early studies using this model, drugs were given prior to spasms induction with NMDA. In a more recent study, ACTH was given after spasms induction, more closely approximating the clinical scenario [80]. This model can be potentially utilized to investigate the brain networks involved in IS initiation and propagation.

#### 2.2.4. Severe Structural Lesions: Multiple-Hit Model

The multiple-hit model might represent symptomatic IS cases, in which an etiology is known. The model is established by intracerebral injection of the antineoplastic agent doxorubicin (DOX), which promotes oxidative damage, plus intracerebroventicular administration of the pro-inflammatory compound lipopolysaccharide (LPS), both on P3, followed by intraperitoneal injection of the tryptophan hydroxylase inhibitor *p*-chlorophenylalanine (PCPA) on P5. DOX and LPS are used to disrupt cortical and subcortical structures and their connections, which might be an obligatory feature of IS, while PCPA reduces the amount of serotonin in the brain as some cases of IS exhibit low CSF serotonin metabolites [81]. Spasms with IS-like EEG characteristics are then observed and they often evolve into other seizure types; cognitive deficits and autism-like behaviors are observed after P9 [82].

ACTH and vigabatrin were tested in this model. Only vigabatrin suppressed the spasms, though it was associated with a high mortality rate [82]. To address this issue, the vigabatrin analog CPP-115, which has a higher affinity for GABA aminotransferase and less retinal toxicity, was attempted. Low doses of CPP-115 reduced spasms without increased mortality, but higher concentrations had similar toxicity as vigabatrin [83]. The effectiveness of vigabatrin and its analogs might be explained by the selective reduction of cortical parvalbumin interneurons seen in histological studies of this model [84].

A high percentage (as many as 40–50%) of patients with tuberous sclerosis complex (TSC) manifest IS [85]. The mechanistic target of rapamycin (mTOR) inhibitor rapamycin can reduce seizures, including IS, in patients with TSC [86], so this compound was trialed in the multiple-hit model; rapamycin suppressed spasms in a dose-dependent fashion and it significantly improved cognitive measures [87].

Other drugs have also been screened using the multiple-hit model. Carisbamate, a broad spectrum ASD thought to act on targets independent of Na^+^ channels, exerted similar positive results, suppressing behavioral and electroclinical spasms [88]. Galanin agonists have been shown in multiple preclinical models to have antiepileptic and neuroprotective characteristics, but acute injections of NAX 5055 (a galanin analog) did not reduce the number or severity of spasms in the multiple-hit model [89]. Similarly, the caspase 1 inhibitor VX-765 (belnacasan), the GABA_B_ receptor inhibitor CGP35348, and multiple injections of 17β-estradiol did not have any effect on spasms [90]. The rationale for testing belnacasan was that it would counteract the pro-inflammatory effects of LPS by inhibiting interleukin-1β (IL-1β) production, which has been shown to have antiseizure properties in temporal lobe epilepsy models [91].

The significant brain insults that were caused by the combination of toxins used to establish this model (DOX, LPS, PCPA) would most closely mimic very severe cases of symptomatic IS. It remains a challenge to investigate potential treatments in less extreme cases of symptomatic IS. A major benefit of the multiple-hit model is the ease of screening proposed anti-IS compounds, although none of the many tested drugs have yet reached clinical use.

## 3. Pathophysiology of EEG Patterns in Infantile Spasms

The physiological basis of the main EEG patterns seen in IS—interictal hypsarrhythmia and ictal electrodecrement—is completely unknown and has not received much attention from researchers until recently. This knowledge gap, in part, relates to species differences (rodents and other animal models do not closely approximate the incredible complexity of the human brain, and these species differences are reflected in disparate EEG patterns) and, in part, to the inherently complicated problem of trying to sort out cellular correlates from scalp-recorded electrographic patterns. Yet, the elucidation of this intractable question would provide valuable insights into the pathophysiology of IS and even point to novel therapeutic targets.

**Figure 2:**
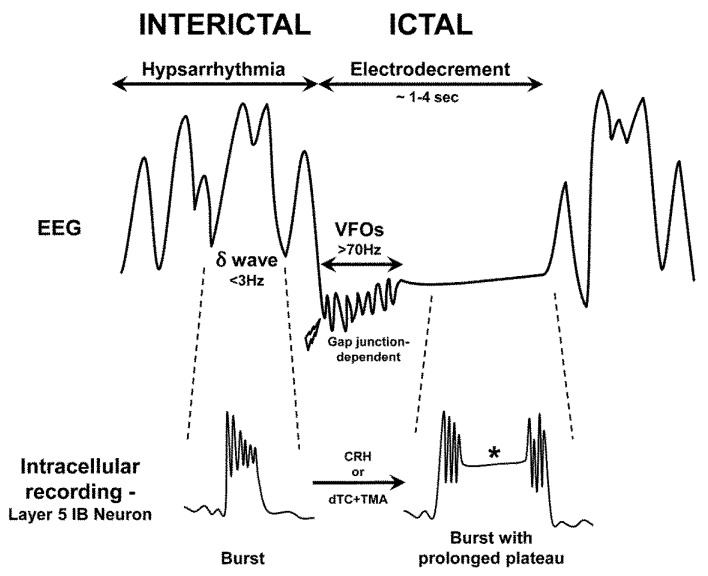
Schematic showing characteristic electroencephalogram (EEG) findings in infantile spasms. Top trace: Hypsarrhythmia (interictal) consists of chaotic, high-voltage irregular slow waves (delta range, δ, <3 Hz) with superimposed sharp waves and spikes. Lightning bolt indicates onset of a clinical spasm. The EEG sometimes exhibits initial very high frequency oscillations (VFOs, >70 Hz) that are mediated by gap junctions, followed by electrodecrement (attenuation of voltage during the clinical spasm). Once the spasm ends, the electrodecrement ceases and hypsarrhythmia resumes. Bottom trace: The cellular correlates of the different phases of the EEG are indicated. During hypsarrhythmia, layer 5 pyramidal neurons (intrinsic bursting (IB) type) fire in bursts, accompanying each EEG delta wave. These bursts require N-methyl-D-aspartate (NMDA) receptors and GABA_B_ receptors. During electrodecrement, delta waves are suppressed and layer 5 IB neurons are further depolarized, leading to prolonged plateau potentials (asterisk). These plateaus are maximized by an intracellular alkaline pH and involve glutamate release and increased Ca^2+^ influx. The transition from interictal to ictal firing can be experimentally induced by the endogenous proconvulsant corticotropin-releasing hormone (CRH) or exposure to the combination of d-tubocurarine (dTC, an acetylcholine receptor antagonist) and trimethylamine (TMA, an alkalinzing agent that enhances gap junction opening). Though simplified, this scheme illustrates potential targets for novel therapeutics (see text).

In this regard, an elegant review synthesizes recent experimental and modeling data to provide plausible, experimentally testable explanations for hypsarrhythmia and electrodecrement [4]. These ideas are summarized below and in Figure 2.

Hypsarrhythmia is a very chaotic EEG pattern that is composed of extremely high amplitude (>200 microvolts), irregular (not rhythmic) slow waves (in the delta range, <3 Hz) with intermixed sharp waves and spikes. In a child with IS, hypsarrhythmia predominates the EEG and it has been implicated in the encephalopathy and developmental regression seen in this syndrome. That is, hypsarrhythmia occupies the vast majority of the EEG recording, while the actual seizures (spasms with associated electrodecrement) only comprise a small fraction of total time. In IS, the therapeutic goal is to eliminate both the ictal spasms and the interictal hypsarrhythmia (the latter is probably responsible for the cognitive and developmental declines).

The cellular basis of the irregular slow waves that are characteristic of hypsarrhythmia is hypothesized to reflect activity in a particular subtype of neocortical layer 5 neurons, called intrinsic bursters (IBs) [94]. IBs are the source of normal cortical delta waves, such as those seen during deep sleep, when cholinergic excitation is low and dopaminergic tone is almost nil [94]. Bursting activity in these IB neurons is dependent on functional NMDA- and GABAB-receptors. Two experimental manipulations, when combined, disrupt IB periodic firing and produce a hypsarrhythmia-like pattern: (1) intracellular alkalinization with trimethylamine (TMA)) and (2) removing the excitation of a major subset of early-developing interneurons by blocking acetylcholine receptors [4]. This treatment disinhibits the cerebral cortex, enhances glutamate release, and markedly increases the magnitude of both the IB neuron burst and initial delta rhythms, all favoring a hypsarrhythmia-like pattern. Each burst in layer 5 neurons corresponds to a delta wave recorded on surface EEG or an abnormal slow event in the IS model (Figure 2).

On the other hand, electrodecrement is characterized by the suppression of delta slow waves and attenuation of the EEG background, which becomes nearly flat for several seconds, while the spasm is occurring. The electrodecrement is thought to occur when a sufficient number of layer 5 IBs develop sustained plateau depolarizations (Figure 2) [95]. These plateaus require intact (and probably enhanced) Ca^2+^ conductances and an alkaline cystolic pH. An alkaline intracellular pH, as with TMA exposure, has multiple effects on neurons, including an enhancement of Ca^2+^ conductances, increased glutamate release, and opening of gap junction channels [4]. TMA also blocks K^+^ conductances. Excessive Ca^2+^ influx into IB neurons could be excitotoxic and contribute to the encephalopathy in IS. The requirement of an alkaline intracellular pH is intriguing, in that drugs that are mildly acidic in nature, such as acetazolamide and topiramate, are sometimes successful in suppressing IS [96,97]. Simultaneous measurements of neuronal electrical activity and pH will be necessary to further sort out these mechanisms. These observations also raise the possibility that the blockers of Ca^2+^ conductances could be anticonvulsant or even neuroprotective in IS. Of note, CRH increases Ca^2+^ conductance and addition of CRH to neocortical slices results in facilitated burst discharges and plateau potentials [98]. Therefore, as a potent convulsant in early brain development, CRH facilitates neuronal plateaus, further supporting the hypothesis that CRH receptor blockade or Ca^2+^ channel blockade (or both) might be therapeutic in IS. Several currently available agents or metabolic therapies downregulate the CRH receptors (ACTH [54]) or block Ca^2+^ channels (e.g., verapamil [99]—although verapamil has variable and limited effectiveness as an antiseizure drug; fructose-1,6-biphosphate [100]). Calcium channel blockers seem ripe for investigation in such models.

Very fast EEG oscillations (VFOs, >70 Hz) are sometimes seen during the early part of the ictal electrodecrement (Figure 2). Intracellular alkalinization enhances VFOs and they likely reflect activity in neurons in more superficial cortical layers (i.e., layers 2 and 3), widely transmitted by gap junctions between cortical pyramidal neurons, as these potentials are blocked by gap junction inhibitors [101]. Gap junctions represent an attractive mechanism for the rapid activation of neuronal networks and they are especially prominent early in development [102].

It might be envisioned that IS-associated EEG changes result from an entire cortical network of bursting pyramidal cells, intermittently interrupted by brief suppressions of these bursts by an alkaline intracellular pH and other factors, producing the ictal component. The transition from baseline hypsarrhythmia to ictal electrodecrement and then back to hypsarrhythmia remains unexplained by extant data, but the groundbreaking ideas and data of Traub, Whittington, and colleagues allows for numerous pathophysiological hypotheses to be tested.

## 4. Concluding Remarks

Each IS preclinical model has strengths and drawbacks, but, in aggregate, these animal models can be used to further our understanding of this catastrophic disorder and improve the treatment options. The complexity of the pathogenesis of IS renders it virtually impossible to replicate all of the features in an animal model, especially while considering the major differences between the human and rodent brain. Instead, research should focus on developing targeted strategies to investigate particular genetic or cellular phenomena that are known to contribute to IS.

More models will undoubtedly be developed, especially those that are related to gene mutations, but clarification of the critical knowledge gaps in IS will require more than just additional models. Each model must be used to rigorously address specific mechanistic questions. This approach will have the highest impact in elucidating the physiological basis of the spasms, IS-associated EEG changes, and the devastating cognitive sequelae of this disorder.

## Figures and Tables

**Table 1:** Criteria for a Pre-clinical (Animal) Model of Infantile Spasms.

	“Ideal” Criteria	Revised Criteria (Minimal/Sufficient)
**Seizure occurrence and semiology**	Spasm-type seizures (generalized, flexion and/or extension) during 1st year equivalent	Seizures during defined window of brain development
	Spasms occur in clusters	
	Spasms occur within relevant age window (mid-first year in humans)	
	Spasms occur during sleep-wake transitions	
**Drug responsiveness**	Similar to humans (ACTH, corticosteroids, vigabatrin)	Similar to humans (ACTH, corticosteroids, vigabatrin)
**Etiology**	Multiple relevant etiologies	Multiple etiologies
**EEG changes**	Similar to humans: interictal hypsarrhythmia, ictal electrodecrement	Distinct interictal and ictal changes
**Cognition, behavior**	Regression	Regression

**Table 2:** Summary of Selected Currently Described Pre-clinical Models of Infantile Spasms.

Model	Species, Induction Method	Pathophysiology	Major Advantage	Major Limitation	Selected References
**Genetic Models**					
*ARX* deletion (knockout)	Mouse: Deletion of *ARX* from cortical GABAergic interneurons	↓GABAergic interneurons	Relevant to human *ARX* mutation; males more affected	Spasms only in adult mice	[92]
*ARX* expansion (knock-in)	Mouse: Expansion of poly-alanine tract in *ARX* gene, causing interneuronopathy	↓GABAergic interneurons	Mimics known human *ARX* mutation; spontaneous spasms and other seizures later	No hypsarrhythmia	[35]
Ts65Dn mice	Mouse: GABA-B receptor agonist i.p.	Overexpression of GIRK2	Mimics human Down syndrome, which has high incidence of IS	Spasms occur late and not spontaneously	[38]
*APC* knockout	Mouse: Deletion of *APC*	↑ β-catenin → ↑ layer 5 glutamatergic synapses	Involves multiple relevant IS-susceptible genes	EEG changes not similar to human; drug effects not yet reported	[48]
**Acquired/Provoked Models**					
CRH/stress	Rat: i.p. or i.c.v. injection of CRH	Variety of “stressors” causes increased release of CRH, which increases neuronal hyperexcitability	CRH is endogenous convulsant in developing brain	Induced limbic seizures; spontaneous not spasms; ACTH is not effective	[53,57]
TTX	Rat: Intracerebral injection of TTX by osmotic mini-pump	↓ cerebral activity	EEG changes are concordant with human patterns	Spasms occur late in brain maturation; unknown why TTX-induced reduction of neuronal activity leads to spasms	[59,64]
Prenatal stress/NMDA	Rat: Prenatal betamethasone or other stressor on E15, i.p. NMDA on P11	NMDA receptor overactivation	Mimics human cryptogenic IS	Efficacious drug treatments (ACTH, VGB) are given before spasms induction	[66,93,56]
Multiple hit	Rat: DOX, LPS on P3; PCPA on P5	Severe cortical and subcortical structural brain damage	Mimics human symptomatic IS	ACTH has no effect; toxin vs seizure effects	[82,90]

i.p., intraperitoneal; i.c.v., intracerebroventricular; TTX, tetrodotoxin; ↑, increase; ↓, decrease; →, leads to.
